# Acetylproteomics analyses reveal critical features of lysine-ε-acetylation in Arabidopsis and a role of 14-3-3 protein acetylation in alkaline response

**DOI:** 10.1007/s44154-021-00024-z

**Published:** 2022-01-04

**Authors:** Jianfei Guo, Xiaoqiang Chai, Yuchao Mei, Jiamu Du, Haining Du, Huazhong Shi, Jian-Kang Zhu, Heng Zhang

**Affiliations:** 1 State Key Laboratory of Plant Molecular Genetics, Shanghai Center for Plant Stress Biology, Center for Excellence in Plant Molecular Sciences, Chinese Academy of Sciences, Shanghai, 201602 China; 2 University of Chinese Academy of Sciences, Beijing, 100049 China; 3 Hubei Key Laboratory of Cell Homeostasis, College of Life Sciences, Wuhan University, Wuhan, 430072 China; 4 Department of Biology, Institute of Plant and Food Science, Southern University of Science and Technology, Shenzhen, 518055 Guangdong China; 5 Department of Chemistry and Biochemistry, Texas Tech University, Lubbock, TX USA

**Keywords:** Lysine acetylation, Mass spectrometry, PTM crosstalk, 14-3-3, Alkaline stress

## Abstract

Lysine-ε-acetylation (Kac) is a post-translational modification (PTM) that is critical for metabolic regulation and cell signaling in mammals. However, its prevalence and importance in plants remain to be determined. Employing high-resolution tandem mass spectrometry, we analyzed protein lysine acetylation in five representative Arabidopsis organs with 2 ~ 3 biological replicates per organ. A total of 2887 Kac proteins and 5929 Kac sites were identified. This comprehensive catalog allows us to analyze proteome-wide features of lysine acetylation. We found that Kac proteins tend to be more uniformly expressed in different organs, and the acetylation status exhibits little correlation with the gene expression level, indicating that acetylation is unlikely caused by stochastic processes. Kac preferentially targets evolutionarily conserved proteins and lysine residues, but only a small percentage of Kac proteins are orthologous between rat and Arabidopsis. A large portion of Kac proteins overlap with proteins modified by other PTMs including ubiquitination, SUMOylation and phosphorylation. Although acetylation, ubiquitination and SUMOylation all modify lysine residues, our analyses show that they rarely target the same sites. In addition, we found that “reader” proteins for acetylation and phosphorylation, i.e., bromodomain-containing proteins and GRF (General Regulatory Factor)/14-3-3 proteins, are intensively modified by the two PTMs, suggesting that they are main crosstalk nodes between acetylation and phosphorylation signaling. Analyses of GRF6/14-3-3λ reveal that the Kac level of GRF6 is decreased under alkaline stress, suggesting that acetylation represses plant alkaline response. Indeed, K56ac of GRF6 inhibits its binding to and subsequent activation of the plasma membrane H^+^-ATPase AHA2, leading to hypersensitivity to alkaline stress. These results provide valuable resources for protein acetylation studies in plants and reveal that protein acetylation suppresses phosphorylation output by acetylating GRF/14-3-3 proteins.

**Supplementary Information:**

The online version contains supplementary material available at 10.1007/s44154-021-00024-z.

## Introduction

Lysine-ε-acetylation (Kac) is a conserved post-translational modification (PTM) that has been identified in bacteria, fungi, animals, and plants. Large-scale proteomic studies using mass spectrometry (MS) in mammals typically identify hundreds to thousands of acetylation sites in a particular tissue or cell line (reviewed in (Choudhary et al. 2014)). Lundby et al. utilized 16 different types of rat organs and identified 15,474 acetylation sites in 4541 proteins (Lundby et al. 2012), demonstrating that the number of Kac proteins in mammals is in the same range of phosphoproteins. Similar MS-based approaches also identified protein acetylation in a number of plant species, including Arabidopsis (Finkemeier et al. 2011; Hartl et al. 2017; Konig et al. 2014; Liu et al. 2018; Uhrig et al. 2019; Wu et al. 2011), rice (Li et al. 2018), soybean (Smith-Hammond et al. 2014b), pea (Smith-Hammond et al. 2014a), wheat (Zhang et al. 2016a), strawberry (Fang et al. 2015), *Vitis vinifera* (Liu et al. 2019; Melo-Braga et al. 2012), tea (Xu et al. 2017), princess tree (Cao et al. 2019) and Chinese spruce (Xia et al. 2016). In most of these studies, hundreds of Kac proteins were identified, whereas a recent study using the dimethyl labeling method brings the number of Kac proteins in Arabidopsis seedlings to over 2000 (Liu et al. 2018). In both the mammalian and plant systems, Kac targets proteins involved in basic metabolism, translation, and protein folding. However, the overlap between early proteomics studies in plants is usually low, indicating that each study only captured a small fraction of the lysine acetylome (Xing and Poirier 2012).

Close to 400 acetylated non-histone proteins have been functionally characterized in mammals (Narita et al. 2019). These studies indicate that Kac affects protein function in many ways. Besides the well-known function of histone acetylation in chromatin remodeling, Kac has been shown to modulate protein stability, subcellular localization, protein-protein interaction, enzymatic activity, or crosstalk with other types of PTMs (Choudhary et al. 2014). For example, K373ac of p53 prevents polyubiquitination on the same residue and suppresses subsequent degradation (Gu and Roeder 1997). Lys320 and Lys373 acetylation of p53 respectively prevents and enhances phosphorylation of serines in the N-terminal of p53, which regulates its interaction with coactivators and corepressors (Knights et al. 2006). Acetylation of Lys117 and Lys251 in glyceraldehyde-3-phophate dehydrogenase (GAPDH) enhances its translocation to the nucleus (Ventura et al. 2010). Acetylation of Lys99 and Lys140 of malate dehydrogenase (MDH) in *Escherichia coli* and acetylation of Lys307 of MDH in human cells increase the enzyme activity (Venkat et al. 2017).

In contrast, examples on the function of specific Kac proteins are limited in plants. Kac is important for plant-pathogen interactions. The YopJ family of type III effectors, which are conserved in bacterial pathogens, have protein acetyltransferase activity and acetylate diverse plant proteins to suppress host immune response (Song and Walley 2016; Zhang et al. 2016b). On the other hand, some plant immune receptors contain WRKY domains that can detect YopJ catalyzed acetylation and initiate immune responses (Sarris et al. 2015). Kac was also found to regulate basic metabolism and plant growth. Kac of a few metabolic enzymes, including Rubisco, phosphoglycerate kinase 1 (PGK1) and MDH, represses or promotes the enzymatic activity (Finkemeier et al. 2011). The Arabidopsis sirtuin-like deacetylase SRT1 deacetylates and stabilizes cMyc-Binding Protein 1 (AtMBP-1), a transcriptional repressor of glycolysis- and stress-related genes (Liu et al. 2017a). The OsSRT1 was also found to deacetylate glyceraldehyde-3-phosphate dehydrogenase (GAPDH) and inhibit its migration to nucleus, where it functions as a transcriptional activator (Zhang et al. 2017). HISTONE DEACETYLASE 6 (HDA6) was reported to deacetylate BRASSINOSTEROID-INSENSITIVE 2 (BIN2) on K189 and thus inhibit the kinase activity of BIN2, which is an important negative regulator in brassinosteroid signaling (Hao et al. 2016).

Lysine acetylation in eukaryotes is enzymatically regulated by lysine acetyltransferases (KATs) and lysine deacetylases (KDACs), also known as histone acetyltransferases (HAT) and histone deacetylases (HDACs). The KATs identified so far use acetyl-coenzyme A (acetyl-CoA) as a cofactor, a critical metabolic intermediate. Because the physiological concentration of acetyl-coA is close to the dissociation constant between acetyl-CoA and KATs, the level of protein acetylation is affected by cellular acetyl-CoA concentrations (Shi and Tu 2015). Thus, metabolic shifts and energy redistribution sensed by protein acetylation was proposed as important mechanisms to regulate cellular stress response in both animals and plants.

In addition to Kac, plant proteins are subject to other PTMs, such as phosphorylation, ubiquitination and SUMOylation. PTMs are usually interpreted by “reader” proteins that recognize specific modifications. Bromodomain is the main protein module that recognizes Kac, whereas the GRF (GENERAL REGULATORY FACTOR)/14-3-3 proteins are phospho-protein binding proteins present in all eukaryotic cells (Rao et al. 2014). Both bromodomain proteins and 14-3-3 s are ubiquitously expressed in most types of plant tissues and participate in a wide range of biological processes (Lu et al. 2011; Paul et al. 2012). Among the proteins whose activities are regulated by phosphorylation and GRF/14-3-3 proteins, plasma membrane localized proton translocating ATPases (PM H^+^-ATPases) are some of the best characterized. With energy from ATP hydrolysis, the PM H^+^-ATPase pumps protons out of the cell and establishes the pH and electrical potential gradient across the plasma membrane, which is required for cell growth, nutrient uptake and stress responses in plants. In Arabidopsis, AHA1 (AUTOINHIBITED H^+^-ATPASE 1) and AHA2 are the two most abundant PM H^+^-ATPases (Palmgren 2001). AHA1 and AHA2 are essential for plant survival because *aha1 aha2* double mutants are embryo lethal (Haruta et al. 2010). AHA2 contains an autoinhibitory C-terminal region that is subject to phosphorylation and subsequent 14-3-3 binding. The binding of 14-3-3 proteins to the phosphorylated C-terminal region of AHA2 is required to release the inhibitory effects and activates AHA2 activity (Palmgren 2001). Disturbing the interaction between 14-3-3 s and AHA2 results in high sensitivity to high pH and salinity stress.

In this study, we used high-resolution tandem mass spectrometry to profile the lysine acetylome in five representative plant organs, including leaves, roots, stems, flowers and seeds from Arabidopsis. This acetylome exhibits significant overlap with published acetylome datasets and expands the existing lists of Kac proteins and Kac sites by 49% and 62% respectively. Kac targeted proteins are enriched in protein translation and folding, basic metabolism, abiotic stress response, as well as organ-specific processes. In addition, Kac preferentially targets conserved proteins and conserved lysines but most acetylation proteins are not orthologous between plants and animals. We also found that Kac targets proteins largely overlapped with other PTMs, including ubiquitination (Ub), SUMOylation (SUMO) and phosphorylation (Ph). Although Kac, SUMO and Ub all modify lysine residues, they rarely target the same sites. In contrast, Kac sites are surrounded by a significantly higher ratio of phosphorylation sites compared to the background level. Domain analyses indicate that the readers, instead of enzymes, for protein phosphorylation and acetylation, including bromodomain containing proteins and GRF/14-3-3 proteins, are heavily modified by both PTMs, suggesting that they are converging points of acetylation and phosphorylation signaling. Furthermore, we demonstrate that the Lys56 acetylation of GRF6 (14-3-3λ) inhibits its activation of the H^+^-ATPase AHA2 and negatively regulates alkaline stress response in Arabidopsis. Together, our data revealed critical features of the lysine acetylome in plants and serve as an important resource for functional Kac studies.

## Results

### A comprehensive catalog of Kac proteins in Arabidopsis organs

**Fig. 1:**
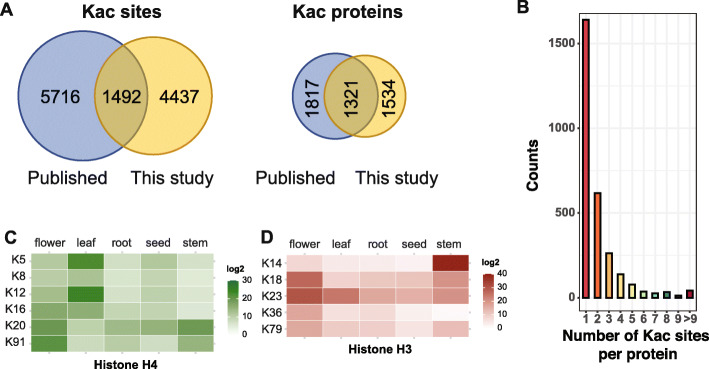
Validation of the acetylome identified in this study. **A.** Comparison of the identified lysine acetylation sites and acetylated proteins from this study with the published datasets (Hartl et al. 2017; Liu et al. 2018; Uhrig et al. 2019). **B.** Distribution of acetylation sites per protein. **C.** and **D.** Quantification of the acetylation sites of histone H4 (**C**) and histone H3 (**D**) in different organs

We developed an acetylproteomic analysis pipeline that includes total protein extraction, trypsin digestion, antibody based Kac peptides enrichment, and high-resolution tandem mass spectrometry (MS/MS). To develop the pipeline, we tested two different protocols for total protein extraction and found that the phenol extraction method produced more Kac peptides than trichloroacetic acid (TCA) extraction method (Supplemental Fig. 1A)(Isaacson et al. 2006). Different combinations of anti-acetyl-lysine antibody and the immunoprecipitation buffer also have significantly different effects on the number of Kac peptides identified (Supplemental Fig. 1B). With this optimized pipeline, we examined the lysine acetylome in five typical plant organs with 2–3 biological replicates per organ, including roots from 2-week-old seedlings, leaves and stems from 5-week-old plants, flowers from 7-week-old plants, and mature seeds (see Materials and Methods for details). The raw MS data were searched against the predicted Arabidopsis proteome (TAIR10) and a decoy database with false discovery rate (FDR) cutoff at 0.01 (Supplemental Fig. 1C). The overlap ratio of Kac sites between any two biological replicates of the same organ was between 71% and 80%, indicating a reproducible detection of the acetylated peptides (Supplemental Fig. 2). In total, we identified 5929 Kac sites from 8284 unique acetyl-peptides belonging to 2887 protein groups (Fig. 1A; Supplemental Table 1 and 2). Each acetylated protein contains ~ 2 Kac sites on average. When we summarized the number of Kac sites per protein, we found the number of proteins containing a certain number of Kac sites decrease exponentially as the number of Kac sites per protein increases (Fig. 1B). This pattern is similar to the observed pattern of compiled phosphorylation sites in human and Arabidopsis (Hu et al. 2015; Zulawski et al. 2013).

In order to validate the acetylome data, we compared acetylation sites of the core histones identified in this study to those in the literature (Shahbazian and Grunstein 2007). Acetylation sites on histone H3 and H4 are extensively studied in plants. Arabidopsis contains one histone H4 protein encoded by 8 different genes. Our dataset contains 7 acetylation sites in histone H4. In addition to the reported acetylation on K5, K8, K12 and K16 of H4 (Zhang et al. 2007), we also identified acetylation on K20, K31 and K91 (Supplemental Table 1). H4K20ac was recently identified in human cells and was associated with transcription repression (Kaimori et al. 2016). H4K31ac was identified in yeast, mouse, human and the parasite *Toxoplasma gondii* (Garcia et al. 2007; Jeffers and Sullivan Jr. 2012; Sundar et al. 2014) and was reported to be present with low abundance in histones associated with the Asf1b complex in human cells (Jasencakova et al. 2010). H4K91ac is located in the histone fold domain and has a role in chromatin assembly in human cells (Ye et al. 2005). Using normalized areas of corresponding spectrums as estimates of relative abundance among different acetylation sites (Supplemental Table 3), H4K31ac cannot be quantified while the other 6 Kac sites exhibited organ specific patterns (Fig. 1C). For example, acetylation of K5 and K12 is more abundant in leaves than in other organs while acetylation of K91 is most abundant in flowers (Fig. 1C).

We also identified two novel acetylation sites in histone H3. Histone variants H3.1 and H3.3 are the two major isoforms of histone H3 in plants, which are associated with transcriptional repression and activation respectively. Because the primary sequences of H3.1 and H3.3 differ by only 4 amino acids, we could not determine whether the majority of acetylation sites occur exclusively in H3.3 except for K36ac and K37ac. Except for K4, every lysine residue at the N-terminal tail (AA 1–40) of histone H3 is acetylated (Supplemental Table 1). K37ac and K79ac have not been reported in plants (Johnson et al. 2004; Kim et al. 2008; Mahrez et al. 2016; Xu et al. 2012; Zhang et al. 2007). H3K37ac was detected in sodium-butyrate-treated human cells (Basu et al. 2009). H3K79ac was also identified in human histone H3 (Garcia et al. 2007). In animals and fungi, methylation of H3K79 is associated with transcriptional activation and DNA repair (Nguyen and Zhang 2011; van Leeuwen et al. 2002). We also identified acetylation sites in other histone H3 isoforms. For instance, we identified acetylation on K9, K14, K18 and K23 in the male-gamete-specific histone H3 (AT1G19890) in flowers, and K87ac in the centromere-specific histone H3 (AT1G01370) (Supplemental Table 1). Quantification of different acetylation sites on H3 also revealed interesting patterns. H3K14ac is most abundant in stems, while K18ac and K23ac are preferred in flowers (Fig. 1D; Supplemental Table 3).

We further compared the acetylation sites in this study against the datasets from three recent acetylproteomics studies (Hartl et al. 2017; Liu et al. 2018; Uhrig et al. 2019). The 3 datasets share 316 Kac proteins and 181 Kac sites (Supplemental Fig. 3). This study covers 80.0% (253/316) of the common Kac proteins and 61.9% (112/181) of the common Kac sites, indicating high coverage the most consistent Kac proteins/sites (Supplemental Fig. 3). It is important to note that all these studies used different plant materials and plant growth conditions for the acetylome analysis, which could be the main reason why the apparent overlap ratio is low. For example, the study by Liu et al. used the aerial tissues from 21-day-old ethylene insensitive mutant *ein3 eil1* grown on MS medium with mock- and ethylene-treated conditions (Liu et al. 2018). The *ein3 eil1* acetylome contains 69.3% (3422/4938) and 77.1% (1115/1447) respectively of the total Kac sites and proteins that were not identified in our study (Supplemental Fig. 3).

Overall, our lysine acetylome dataset not only shows significant overlap with known acetylation sites in Arabidopsis, but also provides the largest catalog of Kac sites to date. The newly discovered Kac sites of histones were also identified in other eukaryotic organisms, suggesting conserved functions of these modifications. These results indicate high accuracy and high sensitivity of our Kac identification pipeline.

### Organ specificity of Kac proteins

**Fig. 2:**
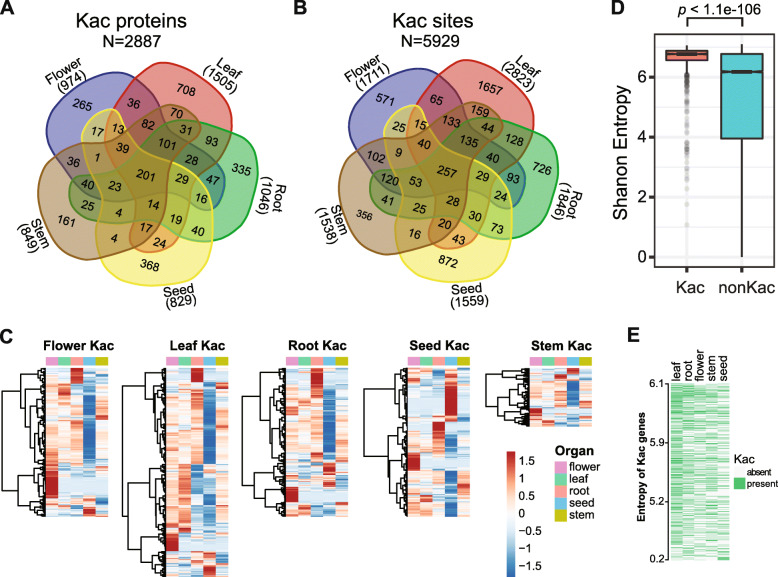
Lysine-ε-acetylation is ubiquitously detected in all 5 tissues of Arabidopsis. **A.** Organ distribution of lysine-ε-acetylation (Kac) sites. **B.** Organ distribution of Kac proteins. **C.** Heatmap showing the mRNA level of tissue-specific Kac proteins in various tissues. **D.** Box plot showing the distribution of Shanon Entropy value of Kac and non-Kac gene expression levels. Width of the boxes indicate variations. **E.** The presence of Kac in different tissues in Shanon Entropy sorted Kac genes

A characteristic difference in development between plants and animals is that plant postembryonic development is constantly modulated by environmental cues (Pikaard and Mittelsten Scheid 2014). To assess organ-specific protein acetylation, we analyzed the distribution of lysine acetylation in five types of Arabidopsis sample. Overall, 201 Kac proteins are shared in these five organs (Fig. 2A), which include core histones, ribosomal proteins, 14-3-3 proteins, protein chaperones and metabolic enzymes (Supplemental Table 1). However, not all the acetylation sites in these proteins are present in all the organs. We detected a total of 998 acetylation sties in these 201 proteins (Supplemental Table 1), but only 257 Kac sites are common in all organs (Figs. 1C and 2B), indicating that the majority of Kac sites are organ-specific.

Between 19% (stem) and 47% (flower) of Kac proteins and between 23% (stem) and 59% (flower) of Kac sites from individual organs are organ-specific (Fig. 2B). To address whether the organ-specific acetylation is due to organ-specific expression, we examined the mRNA levels of the organ-specific Kac proteins in the five different organs utilizing a comprehensive RNA-seq dataset that contains 158 samples from 79 distinct tissues and developmental stages (Klepikova et al. 2016). Seed-specific Kac proteins exhibit the highest level of organ (seed)-specific expression; about 20% of the genes show clear seed-specific expression, including a large number of seed storage proteins (Fig. 2C). In contrast, less portions of other organ-specific Kac proteins correlate with organ-specific expression (Fig. 2C). We further analyzed the expression patterns of the Kac genes by calculating the Shannon Entropy of the genes in the 158 developmental samples (Kadota et al. 2006; Xu and Nussinov 1998). Genes that are expressed at the same level in all samples would have a maximum Shannon Entropy value at 7.3, and a lower value indicates a more organ-specific expression pattern. We found that the Kac genes exhibit significantly higher entropy values than the non-Kac genes (*p* < 1.1e-106, hypergeometric test), suggesting more uniform expression of the Kac genes among different organs than the non-Kac genes analyzed (Fig. 2D). Except for a small number of proteins such as histones, we did not observe a positive correlation between the gene expression uniformity (as measured by entropy) and the protein acetylation patterns in different plant organs (Fig. 2E). Therefore, our results indicate that, compared to non-Kac genes, Kac genes are more uniformly expressed, but acetylation of these proteins are differentially regulated in distinct plant organs.

### Kac targets basic metabolisms and organ-specific processes

**Fig. 3:**
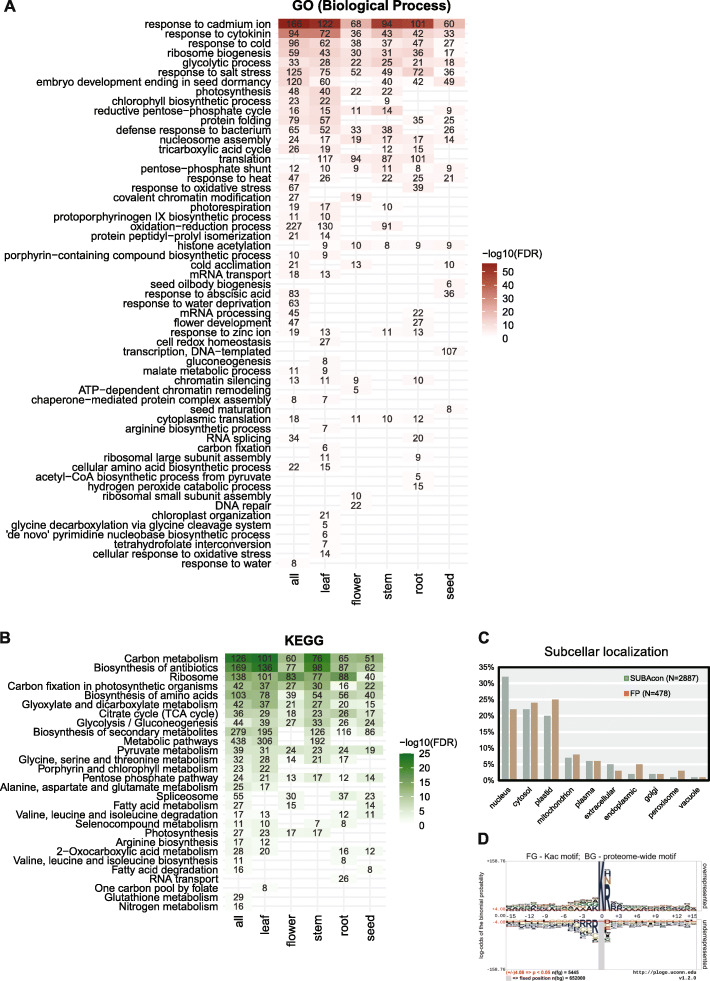
Kac proteins are involved in basic metabolism and tissue-specific processes. **A.** GO (gene ontology) enrichment analysis of identified Kac genes. **B.** KEGG pathway enrichment analysis of all identified lysine acetylation proteins in Arabidopsis. **C.** Subcellular localization of identified Kac proteins. Kac proteins were classified according to SUBA4 localization information. **D.** Sequence logo for all identified Kac sites with all proteins as background population (generated using pLogo)

We next analyzed the Gene Ontology (GO) terms associated with Kac genes. The Biological Process (BP) terms enriched in all the organs can be categorized into several groups, including abiotic stress response, protein translation, and basic metabolic pathways (Fig. 3A). Genes involved in the response to multiple abiotic stresses including cadmium, salt, cold and heat also significantly enriched (Fig. 3A). The role of Kac in cellular metabolic processes was further confirmed by KEGG pathway analysis. Carbon metabolism, biosynthesis of antibiotics and ribosome were the top 3 pathways enriched in all 5 types of samples (Fig. 3B). These results suggest that the changes in acetylation status of metabolic components could alter their activities and thus modulate metabolic flux at critical steps in the relevant pathways. The same analyses also revealed specific processes targeted by lysine acetylation in different organs. For example, Kac Proteins involved in carbon fixation and chloroplast organization were only enriched in leaves while genes involved in seed oil body biogenesis and seed maturation were specifically over-represented in seed Kac genes (Fig. 3A).

We next analyzed the subcellular localization of Kac proteins utilizing the SUBA (Subcellular localization database for Arabidopsis proteins) database (Hooper et al. 2017). SUBA4 contains compiled experimental data using fluorescent proteins (FP) or mass spectrometry (MS), as well as the predicted subcellular localization of the protein using the SUBAcon algorithm (Hooper et al. 2014). Out of the 2855 Kac proteins identified, the SUBA4 database contains 478 proteins with FP evidence in the literature. The analyses using FP and SUBAcon consistently identified nucleus, cytosol, and plastid as the top 3 compartments for Kac proteins (Fig. 3C), each of which contain > 3-fold higher numbers of Kac proteins than any other organelles. The acetylome of mitochondrion has been studied for its central role in energy metabolism in both plants and animals (Hebert et al. 2013; Konig et al. 2014). We identified 213 Kac proteins with predicted mitochondrion localization, which is ~ 35.5% of the number of plastidal Kac proteins (Fig. 3C).

The residues flanking a PTM site usually determine the catalytic preference of enzymes that deposit or remove this modification. We therefore attempted to identify the pattern in motifs associated with Kac sites. We first analyzed the relative frequency of 15 amino acid residues at the N- and C-terminal side of the Kac site using the Arabidopsis proteome as the background (O'Shea et al. 2013). The results indicate that Arg (R), Asn (N), Lys (K), and His (H) are overrepresented at the + 1 position (the 1st residue at the C-terminal side of the acetylated Lys), while negatively charged residues (Glu and Asp), Cys (C), and hydrophobic residues (Leu, Trp, IIe, Tyr etc.) are underrepresented in the + 1 position (Fig. 3D). In general, Lys (K) is overrepresented and Cys (C) is underrepresented in residues surrounding the Kac site (Fig. 3D). Interestingly, Arg (R) is overrepresented in the + 1 to + 3 positions but underrepresented in the − 1 to − 4 positions (Fig. 3D). Because KATs and KDACs are localized in the nucleus and other organelles, we also examined motif patterns in different subcellular compartments. In general, proteins localized to the nucleus, the cytosol, the plastid, and the mitochondrion have similar overrepresented and underrepresented amino acids surrounding Kac sites (Supplemental Fig. 4). The K (ac) R motif is among the most enriched motifs in all 4 compartments (Supplemental Fig. 4).

### Kac targets evolutionarily conserved proteins and conserved Lys

**Fig. 4:**
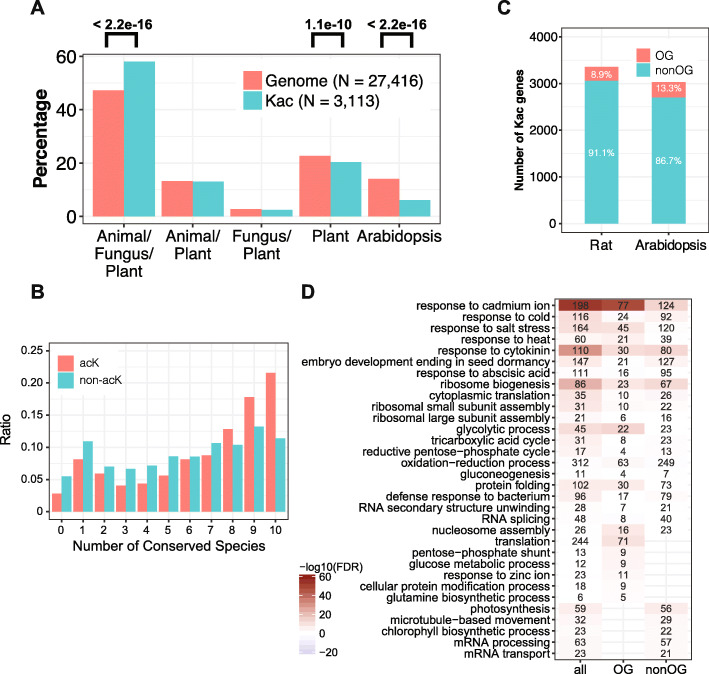
Kac preferentially targets conserved proteins and conserved lysine sites. **A.** The distribution of Kac genes in selected orthologous groups based on the OrthoDB database. **B.** The difference of acK and non-acK in their conserveness in 10 evolutionarily distance plant species. **C.** The number of Kac genes in rat and Arabidopsis and the percentage of genes that belong to the same orthologous group (OG). **D.** GO-enrichment analysis of Arabidopsis Kac genes that are in (OG) or not in (nonOG) the same orthologous group as rat Kac genes

Lysine acetylation has an ancient origin (Nakayasu et al. 2017; Weinert et al. 2013), but whether it is evolutionarily conserved is not clear. To answer this question, we utilized the curated orthologous group (OG) information available in the OrthoDB database that contain genes from > 1000 eukaryotic genomes (Zdobnov et al. 2017). We first asked whether orthologs of Kac proteins can be identified in other species. We found that 58.0% of Kac genes have orthologs in the animal, fungus, and plant kingdoms, whereas on the genome level this ratio is only 47.3% (Fig. 4A). On the other hand, plant- or Arabidopsis-specific genes are significantly underrepresented in Kac genes (Fig. 4A). These data indicate that Kac preferentially targets evolutionarily conserved proteins. We next examined whether Kac modified Lys residues tend to be more conserved. We selected 10 phylogenetically distant plant species (from *Chlamydomonas* to soybean; see Materials and Methods) and identified the closest homolog of Kac proteins from the same orthologous group using reciprocal BLAST. A total of 176 Kac proteins contain close orthologs in the 10 other species. Based on multiple sequence alignments, we found that acetylated Lys (acK) are significantly more conserved (*p* = 7.32x10e-8, Chi Square Test) than non-acetylated Lys (non-acK) (Fig. 4B). About 22% of acK are conserved while only 11% of non-acK are conserved in the 10 selected plant species (Fig. 4B). In support of this observation, 65.6% (3888/5929) of the Kac sites are located within an annotated domain (Supplemental Table 1). We further asked whether the acetylation itself is conserved between different species. We compared our dataset to a published rat acetylome, which contains similar number of Kac proteins from diverse types of organ (4541 proteins from 16 rat organs) (Lundby et al. 2012). We surprisingly found that only 13.3% and 8.9% of Kac genes in Arabidopsis and rat respectively belong to the same orthologous groups (Fig. 4C), although about half of the Kac genes in Arabidopsis contain orthologs in animals (Fig. 4A). Further analyses of enriched GO terms indicated that Arabidopsis Kac genes that are not orthologous to rat Kac genes contain plant-specific processes like photosynthesis and chlorophyll biosynthesis, as well as processes that diverged early during evolution, including microtubule-based movement, mRNA processing and transport (Fig. 4D). However, OG and nonOG Kac genes also target a significant number of overlapped processes (Fig. 4D). In summary, the above analyses indicate that lysine acetylation in plants preferentially targets conserved proteins and conserved lysine residues, but acetylation itself changed rapidly during evolution.

### Kac pattern of ribosomal proteins

**Fig. 5:**
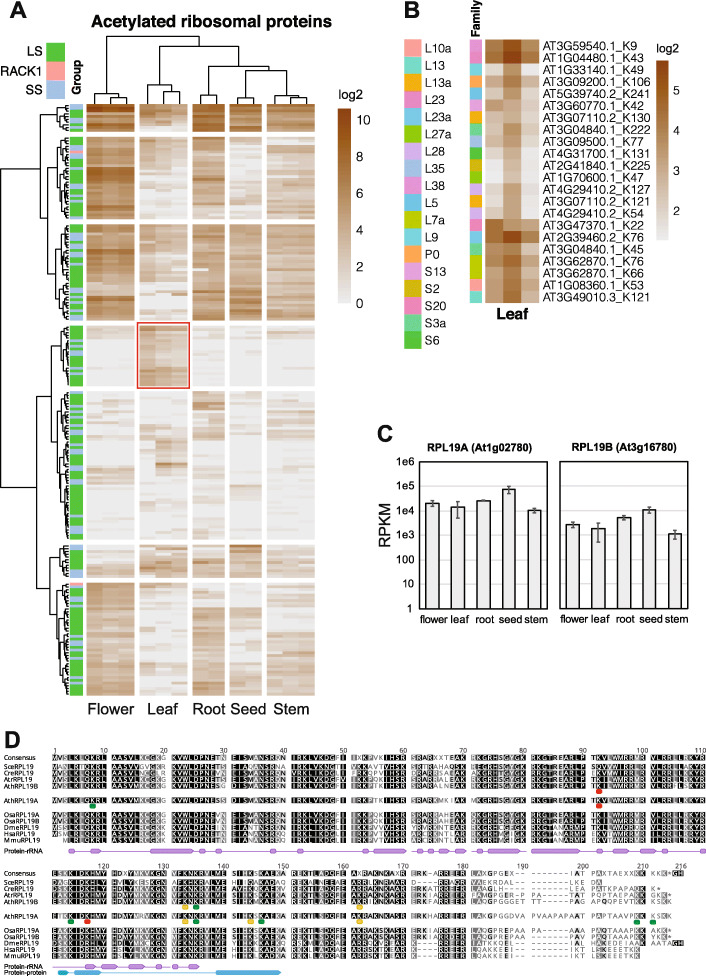
The status of lysine acetylation in ribosomal proteins. **A.** Heatmap of the abundance of ribosomal protein Kac sites in each plant organ. Hierarchical clustering was performed on both the row and the column. The red rectangle indicates leaf specific Kac sites. Color-boxes on the right of the heatmap indicate grouping of ribosomal proteins: LS, large ribosomal subunit; SS, small ribosomal subunit; RACK1, RECEPTOR FOR ACTIVATED KINASE 1. **B.** Zoom in view of the heatmap showing the leaf specific Kac sites. **C.** Transcript expression value of RPL19A and RPL19B in different tissues of Arabidopsis. **D.** Multiple alignment of the L19 subunit of ribosomal proteins from different organisms (*Sce - Saccharomyces cerevisiae, Cre – Chlamydomonas reinhardtii, Atr – Amborella trichopoda, Ath – Arabidopsis thaliana, Osa – Oryza sativa, Dme – Drosophila melanogaster, Hsa – Homo sapiens, Mmu – Mus musculus*). Root-specific Kac sites of Arabidopsis RPL19 proteins are indicated by red dots beneath the sequences. Yellow and green dots respectively indicate other Kac sites that are with or without abundance information. Line-connected purple boxes and skyblue boxes indicate residues at the interface of protein-rRNA and protein-protein interactions within the ribosome

We next performed more detailed analyses on the pattern of lysine acetylation in the cytosolic ribosome because > 60% of cytosolic ribosomal proteins contain lysine acetylation (Supplemental Fig. 5). Different from animals and fungi, most ribosomal protein subunits in plants are encoded by at least two genes. We followed the classification of ribosomal protein genes in a previous study that biochemically characterized protein components of the cytosolic ribosome (Carroll et al. 2008). Hierarchical clustering based on the quantification of acetylation sites classified biological replicates from the same organs into the same groups, indicating the acetylation level of ribosomal proteins are differentially regulated in various organs (Fig. 5A). Clustering also helped visualize organ specific Kac sites (Fig. 5A). For example, 22 Kac sites from 18 different subunit families of ribosomal proteins are leaf specific (Fig. 5B). This organ-specific Kac pattern is unlikely due to differential expression of these ribosomal genes, because transcriptome analyses indicate all of them are ubiquitously expressed in most organs (Klepikova et al. 2016) (see Fig. 5C as an example). Further examination identified other organ-specific Kac sites in the ribosomal subunits. For example, three Lys sites from two L19 subunits are preferentially acetylated in roots (Fig. 5D). Multiple sequence alignment of L19 subunits from plants, yeast (*Saccharomyces cerevisiae*) and animals indicate that almost all 3 root-specific Kac sites are located on the interface of protein-rRNA or protein-protein interactions within the ribosome (Fig. 5D), suggesting that acetylation could negatively affect ribosome assembly. The acetylation sites in RPL19A and RPL19B indicate that these two proteins are differentially regulated by acetylation. While RPL19B-K92ac and RPL19A-K117ac were only detected in the root, RPL19A-K92Ac is also identified at low levels in other organs (Fig. 5D). The alignment also suggests that ribosomal proteins in other organisms are regulated differently. K92 is conserved in plants and animals but changed to Gln (Q) in yeast, which mimics fully acetylated Lys; K117 is conserved in yeast and plants (except for AthRPL19B) but changed to Arg (R) in animals, which mimics non-acetylated Lys (Fig. 5D). RPL19B also has Arg (R) on the 117 positions (Fig. 5D). Except for Lys to Gln or Lys to Arg, no other types of amino acid changes were observed in acetylation sites (Fig. 5D). Overall, these results suggest that Kac is an important modification that regulates ribosome activity during plant development.

### Crosstalk of Kac with other PTMs

**Fig. 6:**
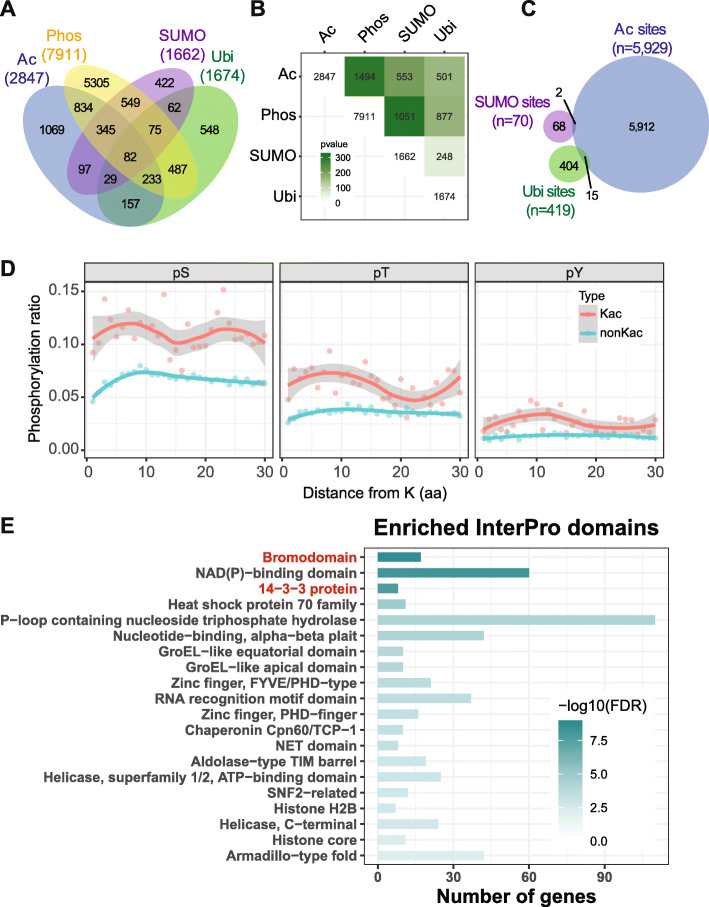
The relationship between lysine acetylation and other PTMs. **A.** Venn diagram showing overlapping proteins among lysine acetylation (Ac), SUMOylation (SUMO), phosphorylation (Phos) and ubiquitination (Ubi) modified proteins. **B.** Calculated *p*-values for the overlaps between PTMs. **C.** Overlap among lysine acetylation sites, ubiquitination sites and SUMOylation sites. Numbers in blocks indicate the number of overlapped genes. **D.** Distribution of phosphorylated serine (pS), phosphorylated threonine (pT) and phosphorylated tyrosine (pY) that are close to the acetylated or non-acetylated lysine. Shaded regions indicate 95% confidence interval. **E.** Enriched InterPro domains in proteins that are targeted by both acetylation and phosphorylation

We examined potential crosstalk of Kac with other PTMs. Phosphorylation is the best characterized PTM, and we compared our dataset to the compiled phosphoproteome data from the PhosPhAt database (Durek et al. 2010; Heazlewood et al. 2008). We also compared our dataset to large-scale ubiquitination (Kim et al. 2013) and SUMOylation studies (Miller et al. 2010; Rytz et al. 2018), because Lys residues can also be modified by these two modifications. We found that Kac proteins largely overlapped with proteins modified by SUMOylation, ubiquitination, or phosphorylation. (Fig. 6A). About 30% of SUMOylated or ubiquitinated proteins are also acetylated (Fig. 6B). However further analyses of the modification sites indicate that only 2 sites are shared between SUMOylation and Kac, and 15 sites are shared between ubiquitination and Kac (Fig. 6C), suggesting that they usually do not modify the same Lys residues. About 52.4% of acetylated proteins are also phosphorylated (Fig. 6A and B). We analyzed the distribution of phosphorylated Ser, Thr, and Try residues surrounding the acetylated Lys (acK). We found that compared to non-acetylated Lys, residues that are close to acK have significantly higher chances to be phosphorylated (Fig. 6D), suggesting that acetylation and phosphorylation interacts locally, or that they prefer to target similar regions of the protein. We attempted to identify the critical nodes where acetylation and phosphorylation signaling crosstalk through analyzing the protein domains that are both acetylated and phosphorylated. We found that reader proteins, but not the enzymes (writers and erasers), are significantly enriched in the list (Fig. 6E). Twelve of the 13 GRF/14-3-3 proteins and 17 of the 29 bromodomain-containing proteins are targeted for both acetylation and phosphorylation (Fig. 6E).

### Acetylation of K56 in GRF6 represses plant alkaline response

**Fig. 7:**
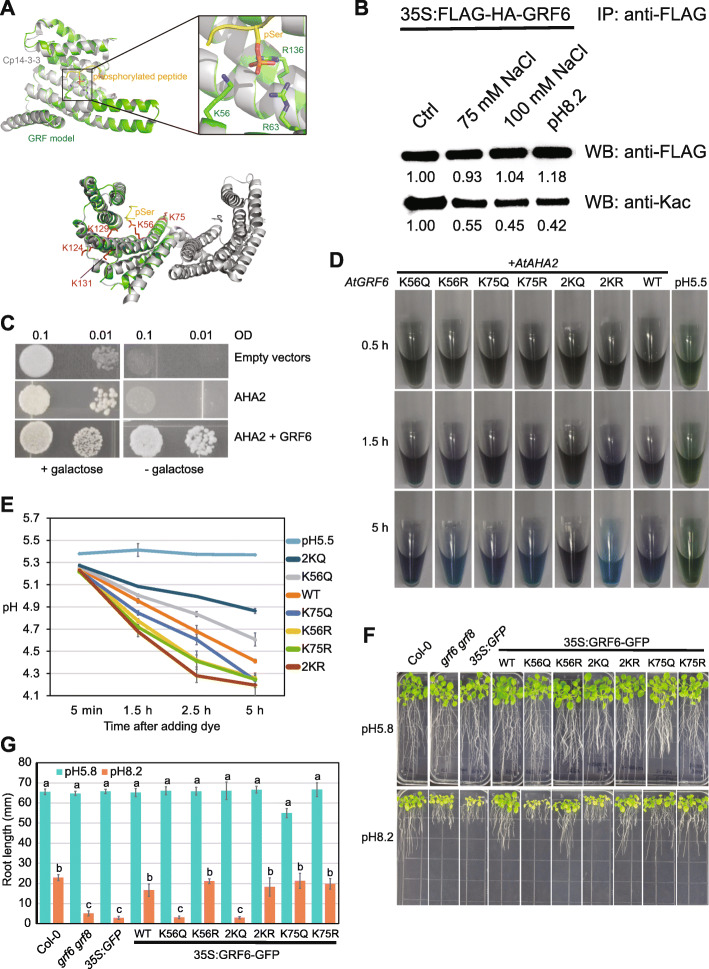
Lys56 acetylation in GRF6 negatively regulates alkaline tolerance in Arabidopsis. **A.** A predicted model of GRF6 structure based on Cp14-3-3. The binding pocket for phosphorylated serine (pSer) that contains 3 positively charged residues (K56, R63 and R136) were indicated in the enlarged panel (top) and the side changes of identified acetylated lysines are indicated in red (bottom). **B.** Western blot showing the protein and acetylation level of immunoprecipitated GRF6. **C.** Drop tests showing that yeast growth required both AtGRF6 and AtAHA2 when glucose was the only carbon source (right panel). The growth of the yeast cells was independent of AtGRF6 and AHA2 when galactose is the sole carbon source (left panel). **D.** Methyl purple staining indicating the pH change of the growth media affected by the expression of AtAHA2 and different version of AtGRF6. **E.** Quantification of the pH change in (**D**). **F.** The root growth phenotype of plants growing on plates with normal (pH 5.8) and high pH (pH 8.2). **G.** Quantification of root lengths in (**F**). The root length was measured using imageJ. Error bars represent standard deviation from 12 seedlings. Significant differences were indicated by different lowercase letters after t-tests (*p* < 0.05)

To understand the possible functions of Kac in 14-3-3 proteins, we performed structure modeling of the GRF6/14-3-3λ protein. The predicted molecular structure of GRF6 is almost identical to the crystal structure of the 14-3-3 protein from *Cryptosporidium parvum* (Brokx et al. 2011) (Fig. 7A). Three positively charged residues, K56, R63, and R136 form a binding pocket for phosphorylated Ser (pSer). We identified 5 acetylation sites (K56, K75, K124, K129, K131) in GRF6, among which K56ac and K75ac are the most abundant (Supplemental Table 1; Supplemental Fig. 6A-B). K75 is outside but spatially close to the pSer binding pocket (Fig. 7A). Because acetylation neutralizes the positive charge on the Lys side chain, we predict that K56ac reduces the binding of GRF6 to phosphorylated proteins. Immunoblot analyses of total 14-3-3 proteins and GRF6 revealed that acetylation levels of 14-3-3 s and GRF6 was significantly reduced by salt stress or alkaline stress (Fig. 7B; Supplemental Fig. 6C). The decreased Kac under stress conditions may facilitate the interaction between GRF6 and its phosphorylated binding partners.

We first tested the above hypothesis using a binary heterologous system in yeast. Binding of 14-3-3 s to phosphorylated Thr947 in AHA2 is required to activate the proton ATPase activity of AHA2 (Palmgren 2001). The yeast strain RS-72, where the promoter of yeast endogenous PM H^+^-ATPase gene (*PMA1*) was replaced by the *GAL1* (galactose inducible) promoter, has been used to study the effect of individual phosphorylation events on the activity of AHA2 (Fuglsang et al. 2007; Palmgren 2001). The yeast genome (*S. cerevisiae*) contains two 14-3-3 genes, *BMH1* and *BMH2*. We modified the RS-72 strain by knocking out the *BMH1* gene and replacing the promoter of *BMH2* with the *GAL1* promoter sequence (Supplemental Fig. 6D). The resulting yeast strain (named RS-72v for RS-72 varied) could grow in the medium that contains galactose, but not glucose, as the carbon source (Fig. 7C). When the PM H^+^-ATPase gene and the 14-3-3 gene from Arabidopsis (*AHA2* and *GRF6*) were transformed into RS-72v, the yeast can then grow on medium that uses either galactose or glucose as the carbon source (Fig. 7C), indicating that the growth of the RS-72v strain on glucose medium relies on the action of AHA2 and GRF6. We next examined the effects of GRF6 acetylation on AHA2 activity in the RS-72v strain. We mutated K56 and K75 to Arg (R) and Gln (Q) respectively to mimic the non-acetylated and fully acetylated states of Lys. The effect of GRF6 Kac on the activity of AHA2 was directly visualized and measured using the pH indicator methyl purple (MP), which is stable in the yeast medium for at least 9 h (Supplemental Fig. 6E). The A530/A430 ratio of MP linearly correlates with pH changes from 4.7 to 5.5 (Supplemental Fig. 6F). When glucose is the sole carbon source in the medium, AHA2 is the main PM H^+^-ATPase that pumps protons into the media, resulting in decreased pH over time (Supplemental Fig. 6D). This effect is evident in the yeast strain transformed with wild type (WT) GRF6; the medium pH decreased from 5.25 to 4.41 over the course of 5 h and the medium color changed from green to light purple (Fig. 7D and E). Consistent with our prediction, K56Q (acK mimicking) reduces and K56R (K mimicking) enhances the AHA2 activity (Fig. 7E). K75Q and K75R of GRF6 decrease the AHA2 activity to different levels but mutating both residues has stronger effects than any single point mutations: yeasts with 2KQ (K56Q K75Q) and 2KR (K56R K75R) have the lowest and highest AHA2 activities respectively (Fig. 7E). As a control, all the different mutants of GRF6 have no detectable effects on the activity of AHA2 when galactose is used in the culture medium (Supplemental Fig. 6G and H). These results strongly support that the acetylation of Lys56 in GRF6 negatively regulates GRF6 binding to and activation of AHA2.

We next examined whether the same effects of GRF6 acetylation could be observed in plants. We generated transgenic Arabidopsis plants overexpressing *GRF6-GFP* and the point mutants (K to R and K to Q) in the *grf6 grf8*/*14-3-3λκ* double mutant background. The *grf6 grf8* plants showed hypersensitivity to alkaline medium (pH 8.2) as reflected by the significantly reduced leaf sizes and root length (Fig. 7F and G**)**. Expression of wild type GRF6 recovered the sensitivity of the mutant, but K56Q or 2KQ mutants of GRF6 could not complement the hypersensitivity phenotype (Fig. 7F and G), indicating that K56 is a major acetylation site in GRF6 that negatively regulates activity of PM H^+^-ATPase in Arabidopsis. Overexpression of K56R, K75Q, K75R and 2KR mutants complemented the hypersensitivity phenotype but their effects were similar to WT GRF6 (Fig. 7F and G). This result is consistent with the positive effects of these mutants in promoting AHA2 activity and that the Kac level in wild type GRF6 is significantly decreased in response to alkaline stress. Altogether, the results above suggest a model that plants decrease K56ac in GRF6 in response to alkaline stress, which in turn enhances the plasma membrane H^+^-ATPase activity and plant fitness.

## Discussion

### Features of the lysine acetylome in plants

Proteins as the major executors in cellular functions are subject to PTMs, which alter properties of proteins in response to various stimuli and are crucial for cellular metabolism, signal transduction, growth and development (Enserink 2015; Inze and De Veylder 2006). The ε amino group of lysine is positively charged under physiological pH and can be used for ionic interactions, for hydrogen bonding, or as a general base in enzyme catalysis. Acetylation neutralizes the positive charge of the ε amino group and introduces steric hindrance; as a result, Kac can affect many aspects of protein function. Because of the central role of acetyl-CoA in basic metabolism, lysine acetylation is believed to provide a mechanistic link between metabolic state and cellular signaling (Choudhary et al. 2014). Plants are fundamentally different from animals in the energy acquisition process. Thus, it is reasonable to assume that the plant acetylome has its unique features.

To date the largest number of acetylated proteins in plants were identified using Cesium chloride (CsCl) density gradient centrifugation-based protein fraction and the in-house developed Stable isotope-based Quantitation-Dimethyl (SQUA-D) software suitable for dimethyl labeling analyses (Liu et al. 2018). Due to low efficiencies in enriching acetylated peptides, it is still challenging to identify acetylated proteins with reasonable coverage of the acetylome. Therefore, we optimized the protocol for the identification of acetylated proteins by combining efficient total protein extraction (Isaacson et al. 2006), and antibody-based acetylpeptide enrichment (Isaacson et al. 2006; Shteynberg et al. 2013; Wu et al. 2013). This protocol allowed us to identify a total of 5929 Kac sites in 2887 proteins in five representative Arabidopsis organs (Supplemental Tables 1 and 2). This comprehensive catalog of Kac sites, together with previously published acetylome dataset, bring the number of Kac sites in plants to the same magnitude as the number of phosphorylation sites.

A few recent studies in mammals suggest that the stoichiometry of lysine acetylation is low at the proteome level (Tatham et al. 2017; Weinert et al. 2015). This raises the concern that some of the acetylation sites are due to stochastic non-enzymatic acetylation, which could be the alternative explanation for limited overlaps between published plant acetylomes (Supplemental Fig. 3) (Xing and Poirier 2012). However, several lines of evidence support that a large portion of Kac sites we identified are enzyme catalyzed. First, we observed little correlation between the expression level of acetylated proteins and their acetylation status in different organs, suggesting that acetylation is not a stochastic event mainly determined by the concentration of the protein and acetyl-CoA (Fig. 2). Second, our results indicate that the nucleus is the organelle that contains the highest number of acetylated proteins in the cell (Fig. 3C). This is consistent with the fact that the majority of KATs and KDACs are localized to the nucleus (Shen et al. 2015). Third, acetylated lysine residues are significantly more conserved than the other lysine residues (Fig. 4), indicating that the acetylation does not occur by chance.

On the other hand, ~ 68% of the Kac proteins are predicted or experimentally verified to be in the other cellular compartments (Fig. 3C). Since only a small number of KATs are located outside the nucleus, it raises the question whether most lysine acetylation outside the nucleus occur through non-enzymatic mechanisms. For example, the large subunit of RuBisCo, RbcL, can be non-enzymatically acetylated in vitro (Gao et al. 2016). Lysine acetylation could be an important mechanism to regulate RuBisCo activity in vivo in response to fluctuations in the cellular energy status. Non-enzymatic acetylation using acetyl-CoA as a reactant is facilitated by high pH, which deprotonate the ε amino group and facilitates its nucleophilic attack on acetyl-CoA. In mammalian cells, the majority of Kac proteins in the mitochondrion are non-enzymatically acetylated with the pH in the matrix around 8 (Wagner and Payne 2013). We also found that mitochondrion localized Kac proteins account for ~ 8% of total acetylated proteins (Fig. 3C). However, high prevalence of Kac proteins in other cellular compartments cannot be explained by the pH alone. The cytosol- and plastid-located Kac proteins are the next two largest groups after the nuclear Kac proteins, but both the plastid stroma and the cytosol have a pH around 7.2 as measured by GFP-based pH sensors (Shen et al. 2013). On the other hand, peroxisomes and vacuoles have a pH of 8.4 and 5.2 respectively (Shen et al. 2013), which are essentially the highest and lowest pH values among all organelles, but they contain similar number of Kac proteins. While the possibility that organelle-localized unknown KATs catalyze the acetylation reaction cannot be ruled out, it is also possible that these proteins are acetylated in the cytosol and then transported to specific organelles, considering that a few KATs are localized in the cytosol or exhibit nucleocytoplasmic shuttling (Shen et al. 2015).

Motif analyses revealed clear preferences of amino acids surrounding Kac sites, which may reflect the preference of KAT and KDAC enzymes in plants. The + 1 position next to the Kac site may be most important for acetylation. All positively charged residues including Arg, Lys and His, as well as Asn, an amino acid resembling the biophysical properties of acetylated lysine, are significantly overrepresented in this position (Fig. 3D). In contrast, Cys is underrepresented in most of the positions surrounding Kac sites (Fig. 3D), though proximal cysteine residues have been shown to promote non-enzymatic lysine acetylation by forming an S-acetylated intermediate with acetyl-CoA, which then transfers the acetyl group to lysine through an S-to-N transfer reaction (Tatham et al. 2017). Negatively charged residues like Asp and Glu are underrepresented in the + 1 positions of Kac sites (Fig. 3D). In principle negatively charged residues could help deprotonate lysine and facilitate the acetylation reaction. In rat, Kac preferentially occurs in Lys-rich regions with negatively charged amino acids surrounding the Kac sites (Lundby et al. 2012). These differences in the motif pattern are consistent with the observed low percentage of orthologous Kac proteins between Arabidopsis and rat (Fig. 4C), even though acetylation-modified proteins and lysines are highly conserved during evolution.

### Protein acetylation in plant development

Here we profiled the protein lysine acetylome in five representative organs of Arabidopsis. The plant materials were collected from different developmental stages. Roots were from 2-week-old seedlings grown on plates, leaves and stems from 5-week-old plants, and flowers from 7-week-old plants. Thus, the data reported in this study only represent snapshots of the highly dynamic acetylome during plant development. More systematic sampling of organs from the same developmental stages (e.g., roots from 5-week-old plants) are needed to reveal system level Kac signaling in the future. While this will likely unveil a more comprehensive lysine acetylome, current data already identified candidates involved in organ specific processes. For example, photosynthesis-related proteins are exclusively enriched in aboveground organs, while the seed oil body biogenesis and seed maturation proteins are acetylated in seeds (Fig. 3A). Quantitative analysis further revealed the potential function of lysine acetylation in house-keeping proteins. Ribosomal proteins (RPs) are among the most conserved groups of proteins in evolution. As the main machinery for protein synthesis in the cell, ribosomes are subject to intense regulations during development or under stress conditions. Different from the yeast and animals, plant RPs are encoded by small gene families containing 2–7 members; mutations in certain RP genes lead to specific developmental phenotypes (reviewed in (Byrne 2009)). We identified lysine acetylation in > 60% of ribosomal proteins (RPs). The acetylation level of RPs alone is sufficient to distinguish samples from different plant organs and a significant portion of the Kac sites are organ specific, suggesting that Kac is one of the mechanisms that regulate ribosome activity during development (Fig. 3B). For example, most of the acetylation sites identified in two L19 subunits are located on the predicted interface of protein-protein interactions or protein-rRNA interactions, suggesting a role of Kac in inhibiting ribosome assembly (Fig. 5D). Given that RPs are rich in positively charged residues that interact with the negatively charged rRNA backbone, this suggests that lysine acetylation could have a general role in inhibiting ribosome assembly.

### Crosstalk between acetylation and phosphorylation

We found that lysine acetylation and other PTMs (phosphorylation, ubiquitination or SUMOylation) modify largely overlapping sets of proteins. This is not surprising because most PTMs target plant metabolism and are important for plant stress response. Acetylation, ubiquitination and SUMOylation all modify lysine residues, but their target sites rarely overlap (Fig. 6C), indicating that they usually do not crosstalk by competing for the same sites. We also found that the “reader” proteins are intensively acetylated and phosphorylated, suggesting that they are crosstalk hotspots. The number of acetylation and phosphorylation sites on GRF/14-3-3 proteins (phosphorylation reader) and bromodomain-containing proteins (acetylation reader) are among the highest of all proteins (Supplemental Table 1). The most highly acetylated lysine in 14-3-3 s forms the phosphor-residue binding pocket with two other positively charged residues (Fig. 7A). We predict that acetylation in this site inhibits the binding of 14-3-3 s to their phosphorylated partners. Indeed, we found that acetyllysine-mimicking mutations (K to Q) reduced the binding of GRF6/14-3-3λ to the PM H^+^-ATPase AHA2, while nonacetylation (lysine)-mimicking mutations (K to R) enhanced the binding of GRF6 to AHA2 (Fig. 7E). Consistently, GRF6 with acetyllysine-mimicking mutations (K to Q) failed to confer alkaline stress resistance in the *grf6 grf8* plants (Fig. 7F). These results support a general model that protein acetylation suppresses phosphorylation output by acetylating 14-3-3 proteins.

## Materials and methods

### Plant materials and growth conditions

The Col-0 ecotype of *Arabidopsis thaliana* plants was used in this study. To collect organs for acetylomics analysis, seeds were surface sterilized and sowed in 0.5x Murashige and Skoog medium (pH 5.8) with 1% (w/v) sucrose and 0.6% (w/v) agar. After 2 days at 4 °C, the plates were transferred to a growth chamber with 16 h–8 h day-night cycle. At the 12th day after sowing, the seedlings were transplanted to soil and grown in a growth room with 16 h–8 h day-night cycle. Leaves (3 rosette leaves and 3 cauline leaves per plant) and stems were collected at the 24th day after transplanting, and flowers were harvested after two additional weeks. To collect roots, surface sterilized seeds were sowed in 0.5x MS medium with 1% (w/v) sucrose and 1.2% (w/v) agar and treated at 4 °C for 2 days. The roots were collected from 14-day-old seedlings grown on vertically placed plates in a growth chamber with 16 h–8 h day-night cycle.

### Protein extraction and in-solution digestion

Total protein from different plant samples was extracted using a phenol-based method as described previously (Isaacson et al. 2006). The precipitated proteins were solubilized in 6 M urea/2 M thiourea/10 mM HEPES buffer (pH 8.0), reduced with dithiothreitol (DTT), and alkylated with iodoacetamide before the digestion by trypsin (Promega). The resulting peptides were purified using the Sep-Pak C18 peptide cartridges (Waters) following standard protocols. For TCA-based protein extraction (Isaacson et al. 2006), plant tissues were ground into fine powder, which was then thoroughly mixed with 10% TCA (v/v) and stored at − 20 °C for overnight. In the next day, total proteins were precipitated, washed twice with ice-cold acetone, and dried in a fume hood. Protein solubilization and trypsin digestion were performed the same as the phenol-based method.

### Affinity enrichment of lysine-acetylated peptides

The enrichment of lysine acetylated peptides was adapted from a published protocol (Wu et al. 2013). Lyophilized peptides were reconstituted in NETN buffer (100 mM NaCl, 1 mM EDTA, 50 mM Tris-HCl, 0.5% NP-40, pH 8.0). The total protein concentration was measured using a Bradford protein assay kit (Bio-Rad 500–0001). For each immunoprecipitation reaction, 5 mg of peptides were incubated with 20 μL of prewashed anti-acetyl-lysine beads (PTM Biolabs Inc., PTM-104) at 4 °C for 4 h with rotation. The beads were then washed for four times in cold NETN buffer and three times in cold pure water. The bound peptides were eluted from the beads with 0.5% trifluoroacetic acid (TFA), and the resulting acetylated peptides were loaded onto prewet Pierce C18 Tips (Thermo Scientific, 87,784) for desalting following the manufacturer’s protocol. The acetylated peptides were lyophilized before downstream analyses.

### LC-MS/MS analysis

Acetyl-lysine enriched peptides were reconstituted in 0.1% Formic Acid (FA) and analyzed on an online nanoAcquity ultraperformance LC (Waters, Milford, MA, USA) coupled with an Orbitrap Fusion Tribrid mass spectrometer (Thermo Fisher Scientific, Watham, MA, USA). Nanospray was controlled by a PicoView Nanospray Source (PV550; New Objective, Woburn, MA, USA) at a spray voltage of 1.9 kV. The peptides were trapped by a 2G-V/MT Trap symmetry C18 column (5 μm particles, 180 μm ID × 20 mm length) at the flow rate of 5 μL/min for 3 min and separated on a BEH130 C18 analytical column (1.7 μm particles, 100 μm ID × 250 mm length) at 250 nL/min. Peptides were eluted with 90 min linear gradient of 3–85% acetone nitrile (ACN) in 0.1% fluoroacetate (FA). Data-dependent MS/MS acquisition was performed following a full MS survey scan by Orbitrap at a resolution of 60,000 over the m/z range of 300–1800 and MS/MS measurements of the top 20 most intense precursor ions. The target values of automatic gain controls (AGC) were set up to 200,000 for Orbitrap MS and 10,000 for ion-trap MS/MS detection. The fragmentations of the selected multiply charged ions were achieved using helium gas and argon at a normalized collision energy of 35% for HCD. Dynamic exclusion was enabled for 60 s. Ions with a charge of 1 or unassigned were excluded from MS/MS analysis.

### Database search

To comprehensively understand the acetyllysine proteomics, two database search strategies were performed, one for identification, one for quantification. For identification, the MS/MS spectra were searched against the Arabidopsis protein database (TAIR10) with a concatenated decoy database supplemented with contaminants using M_ASCOT_ D_AEMON_ 2.4 (Matrix Science, London, UK) software. The resulting data processed with MASCOT-Percolator (Brosch et al. 2009). For quantification, three search algorithms including MS Amanda, Sequest HT, Mascot were applied for spectrum selection, the resulting data were combined using the multi-consensus feature of percolator with consistent FDR in Proteome Discoverer 2.4.0 software (Thermo Fisher Scientific Inc.). For all search tools, Cys carbamidomethylation was set as a fixed modification while Asn/Gln deamidation, Met oxidation and Lys acetylation were set as variable modifications. The enzyme specificity was set to trypsin with the maximum number of missed cleavages set as 4 and maximum number of modifications per peptide set as 5. Peptide-spectrum matches were filtered with the threshold of false discovery rate (FDR) at 0.01 for proteins, peptides, and modification sites. Default parameters were used for other search algorithm or software.

### Plasmid construction and plant transformation

The 14-3-3 λ (GRF6) cDNA fragment was amplified by using RT-PCR and cloned in the pEasy-vector according to manufacture manual (pEASY®-Blunt Zero Cloning Kit), which was used as a template for mutagenesis of GRF6. Site-directed mutagenesis of Lys56 and Lys75 were carried out by PCR amplification with the mutagenic primers (mutations generated are underlined): GRF6^K56Q^, forward primer: 5′-GTAGAGATCCGATCACGTTCTGGTAAGCAACAGAGAGGAGA-3′, reverse primer: 5′-TCTCCTCTCTGTTGCTTACCAGAACGTGATCGGATCTCTAC-3′; GRF6^K56R^, forward primer: 5′-GAGATCCGATCACGTTTCTGTAAGCAACAGAGAGG-3′, reverse primer: 5′-CCTCTCTGTTGCTTACAGAAACGTGATCGGATCTC-3′; GRF6^K75Q^, forward primer: 5′-CGTGTCTTCGATTGAGCAGCAGGAAGAGAGTAGGAAGAAC-3′, reverse primer: 5′-GTTCTTCCTACTCTCTTCCTGCTGCTCAATCGAAGACACG-3′; GRF6^K75R^, forward primer: 5′-CGTGTCTTCGATTGAGCAGAGGGAAGAGAGTAGGAAGAAC-3′, reverse primer: 5′-GTTCTTCCTACTCTCTTCCCTCTGCTCAATCGAAGACACG-3′. The GRF6 wild type and mutated cDNA were cloned into the pCAMBIA 1300-GFP vector containing cauliflower mosaic virus (35S) promoter to generate the 35S:GRF6-GFP and 35S:GRF6m-GFP plasmids (including 35S:GRF6^K56Q^-GFP (carrying a K56Q mutation), 35S:GRF6^K56R^-GFP (carrying a K56R mutation), 35S:GRF6^K75Q^-GFP (carrying a K75Q mutation), 35S:GRF6^K75R^-GFP (carrying a K75R mutation), 35S:GRF6^K56K75Q^-GFP (carrying K56Q and K75Q double mutations), 35S:GRF6^K56K75R^-GFP (carrying K56R and K75R double mutations)). The 35S:GRF6-GFP and 35S:GRF6m-GFP constructs were transformed into the Arabidopsis *14-3-3λ/κ* double mutant (a gift from Dr. Shuhua Yang of China Agricultural University) (Liu et al. 2017b) plants by floral dip method. The T3 or T4 homozygous transgenic plants were used in this study.

### Yeast assays

For yeast assays, the CDS of GRF6 was PCR amplified and cloned into the pEasy vector. Point mutations were generated using the QuickChange II kit (Agilent). The GRF6 fragments were then transferred to the modified pRS415 vector (pRS415 with the yeast ADH1 promoter sequence) to generate pRS415-pADH1:GRF6, pRS415-pADH1:GRF6^K56Q^ (carrying a K56Q mutation), pRS415-ADH1:GRF6^K56R^ (carrying a K56R mutation), pRS415-ADH1:GRF6^K75Q^ (carrying a K75Q mutation), pRS415-ADH1:GRF6^K75R^ (carrying a K75R mutation), pRS415-ADH1:GRF6^K56K75Q^ (carrying K56Q and K75Q double mutations), pRS415-ADH1:GRF6^K56K75R^ (carrying K56R and K75R double mutations). The cDNA of *AHA2* was amplified by PCR and cloned into the vector pRS416 to create *pRS416-pAHD1:AHA2*. The wild-type or mutant *GRF6* vectors were co-transformed with *pRS416-pAHD1:AHA2* into the modified *S. cerevisiae* strain RS-72-v (*MATa ade1–100 his4–519 leu2–3312, ura3*) (Fuglsang et al. 2007), where the promoter of *BMH2* and PMA1 replaced by pGAL1, and BMH1 and URA3 genes knocked out in the RS-72 strain. SC/−Lue, SC/−Ura and SC/−Lue/−Ura media supplemented with glucose or galactose were used to examine the effects of GRF6 acetylation on AHA2 activity. Each experiment was independently replicated more than three times, and each replicate contained yeast cells from three independent transformation events.

### Immunoprecipitation assay

Total proteins were extracted from about two gram of Arabidopsis seedlings in cold IP buffer (50-mM Tris-Cl pH 7.6, 150-mM NaCl, 5-mM MgCl_2_, 10% (v/v) glycerol, 0.1% (v/v) NP-40, 0.5-mM DTT, and 1% protease inhibitor cocktail (sigma)). After cellular debris was removed by centrifugation, the supernatant was incubated with anti-14-3-3 antibody (ARS-AS12 2119, Agrisera) preincubated with Dynabeads (10003D, Invitrogen) or anti-GFP-Trap Magnetic Agarose (GTMA-20, Chrome Tek) at 4 °C for 3 h, and then the beads were washed for four times with IP buffer followed by two times wash with 1x PBS. The immunoprecipitated proteins were analyzed by Western blotting using anti-14-3-3 (ARS-AS12 2119, Agrisera), anti-GFP (D110008, Sangon Biotech) and anti-Actin (D110007, Sangon Biotech) antibodies.

### Plant alkaline sensitivity assay

Five-day-old seedlings grown on 0.5x MS medium with 1% sucrose and 1.2% phytagel (sigma) at pH 5.8 were transferred to 0.5x MS medium of pH 5.8 or pH 8.2 with 1% sucrose and 1.2% phytagel and grow vertically for two more weeks. All the seedlings were placed in a growth chamber at 22 °C under a 16 h–8 h day-night photoperiod. The root length of seedlings was measured in ImageJ.

### Bioinformatics analyses

Gene Ontology (GO) annotation of the identified acetylated proteins was derived from AgriGOv2 database (http://systemsbiology.cau.edu.cn/agriGOv2/). Domain functional description of the identified acetylation proteins was annotated by InterPro domain database (http://www.ebi.ac.uk/interpro/). KEGG pathway was annotated using Kyoto Encyclopedia of Genes and Genomes (KEGG) database (Kanehisa et al. 2004). The subcellular localization of the identified Kac protein was predicted with SUBA4 (http://suba.live/).. The sequence motif was analyzed by pLogo (O'Shea et al. 2013) and Motif-x (Chou and Schwartz 2011). Ten plant species (*Arabidopsis thaliana*, *Brassica oleracea*, *Glycine max*, *Medicago truncatula*, *Zea mays*, *Oryza sativa Japonica*, *Solanum lycopersicum*, *Physcomitrella patens, Selaginella moellendorffii*, *Chlamydomonas reinhardtii*, *Vitis vinifera*) were selected from OrthoDB for the analysis of evolutionary divergence (Waterhouse et al. 2013). Intermediate data processing was performed using in-house developed Perl scripts. All the tile plots, boxplots, and bar plots were generated using the ggplto2 package from R; heatmaps were generated using the pheatmap package from R.

## Supplementary Information

**Additional file 1.**

**Additional file 2.**

**Additional file 3.**

**Additional file 4.**

## Data Availability

The mass spectrometry proteomics data have been deposited in the ProteomeXchange Consortium via the MassIVE partner repository with the dataset identifier MSV000088454.
