# Supplementary material for: Acetylproteomics analyses reveal critical features of lysine-ε-acetylation in Arabidopsis and a role of 14-3-3 protein acetylation in alkaline response
Source: Stress Biol. 2022 Jan 4;2(1):1. doi: 10.1007/s44154-021-00024-z (PMC10442023; doi:10.1007/s44154-021-00024-z)
Supplement: Supplementary file 1 — Additional file 1. [file 44154_2021_24_MOESM1_ESM.pdf]

A

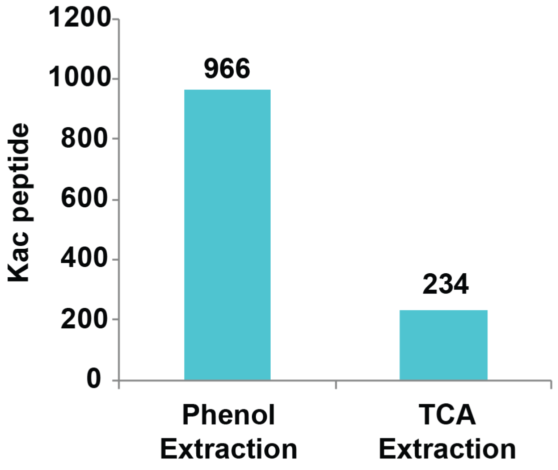

B

	Antibody 1 + Buffer 1	Antibody 1 + Buffer 2	Antibody 2 + Buffer 1	Antibody 2 + Buffer 2
Total peptides	5922	6833	3093	3182
Kac peptides	1433	2223	1122	1351
Enriched ratio	24.20%	32.53%	36.30%	42.50%

C

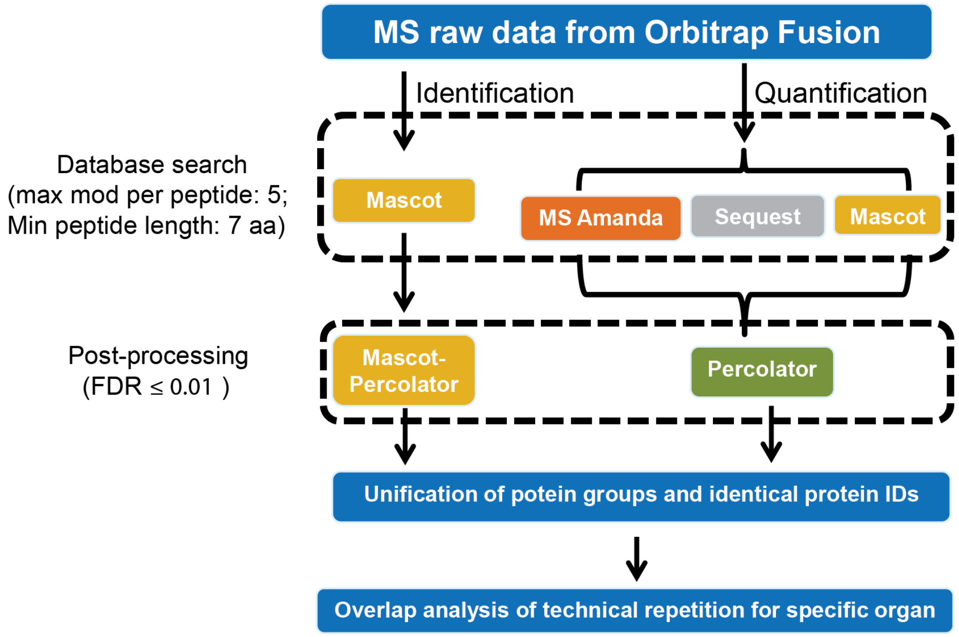

Supplemental Figure 1 Optimization of experiment parameters for the acetylproteomic analysis pipeline. (**A**) Comparison of the number of Kac peptides identified using two different protocols for total protein extraction from (Isaacson et al., 2016) (**B**) Comparison of different combinations of anti-acetyl-lysine antibody and the immunoprecipitation buffer. Antibody 1: Cell Signaling, #9441; Antibody 2: PTM Biolabs, PM104; Buffer 1: 50 mM MOPS pH 7.2, 10 mM Sodium phosphate, 50 mM Sodium chloride (Choudhary et al., 2009); Buffer 2: NETN buffer. (**C**) A flowchart showing data processing procedure after LC-MS/MS.

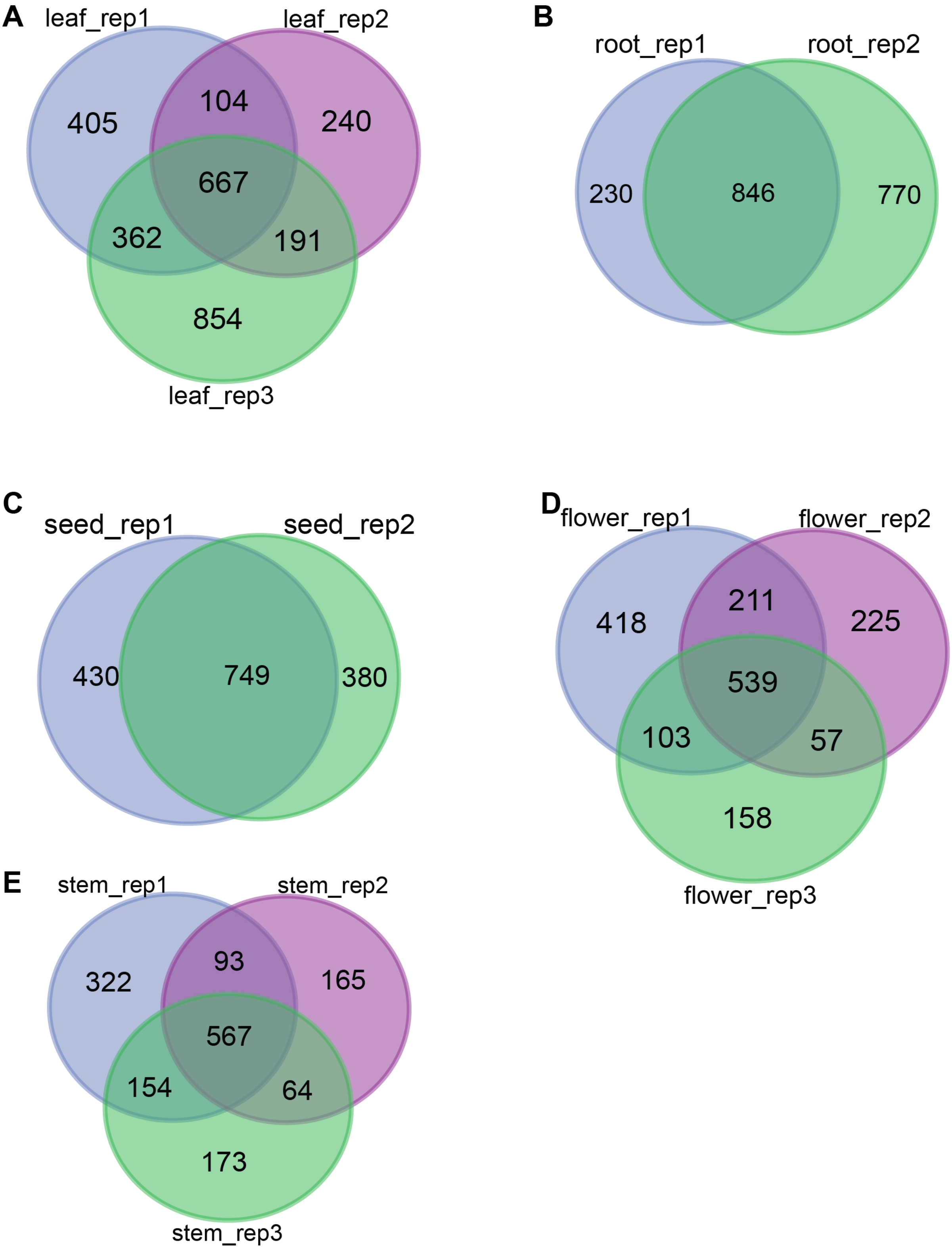

Supplemental Figure 2 Overlap of Kac sites identified between different biological replicates from the same tissue. (A) Leaf. (B) Root. (C) Seed. (D) Flower. (E) Stem.

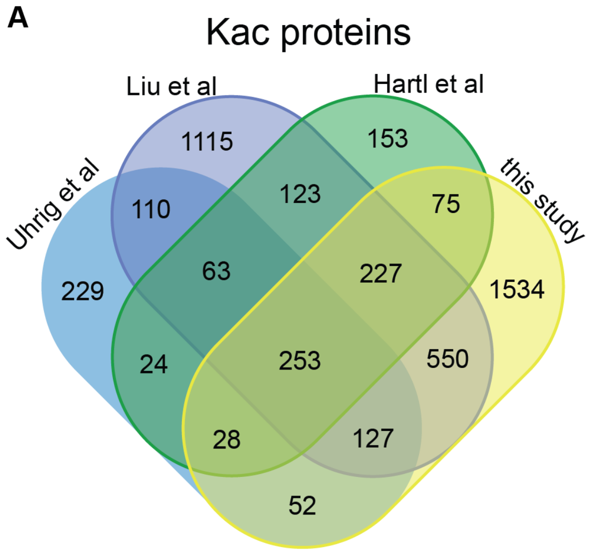

Supplemental Figure 3 Comparison of lysine acetylation sites (**A**) and acetylated proteins (**B**) identified in this study with previous published datasets (Hartl et al, 2017; Liu et al, 2018; Uhrig et al, 2019).

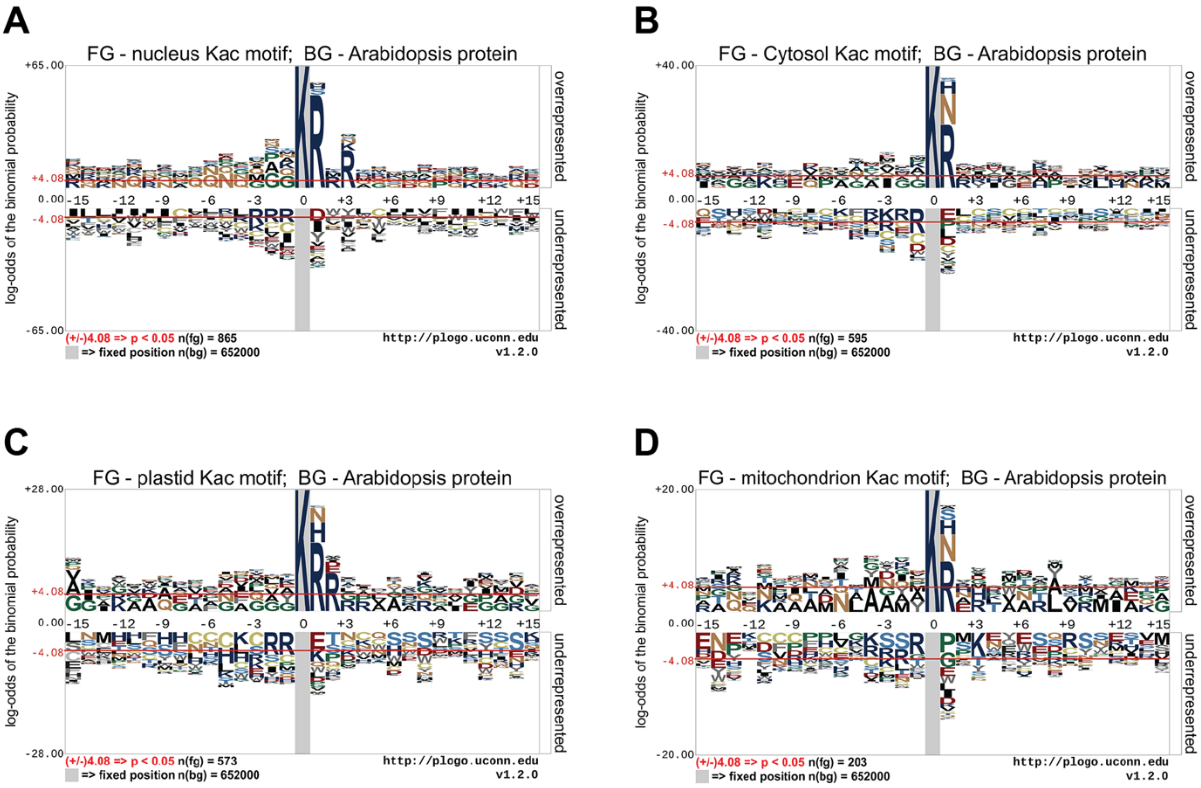

Supplemental Figure 4 Analyses of overrepresented motifs surrounding the acetylation sites of Kac proteins from different organelles. (**A**) Nuclear proteins. (**B**) Cytosolic proteins. (**C**) Plastidal proteins. (**D**) Mitochondria proteins.

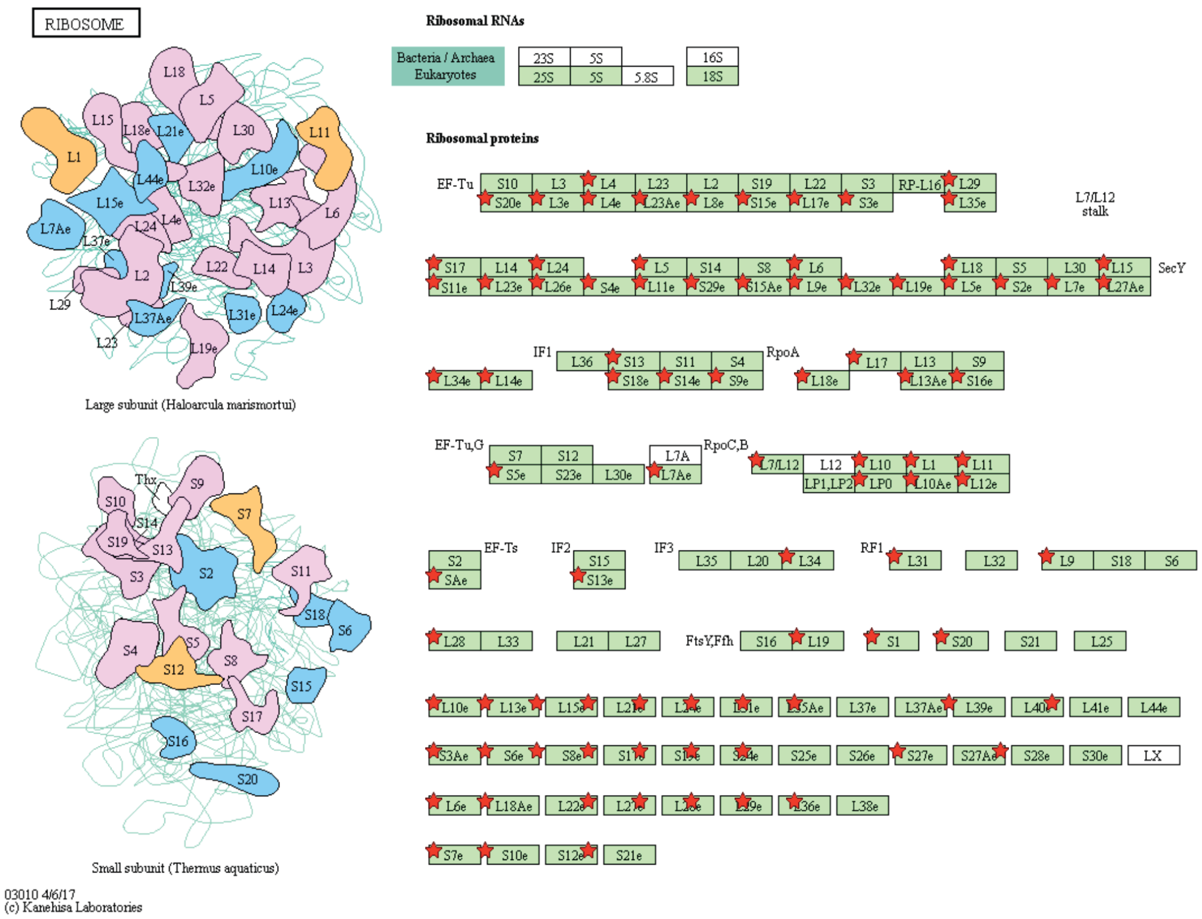

Supplemental Figure 5 Overview of lysine acetylated proteins present in the ribosome. Red stars indicate acetylated proteins.

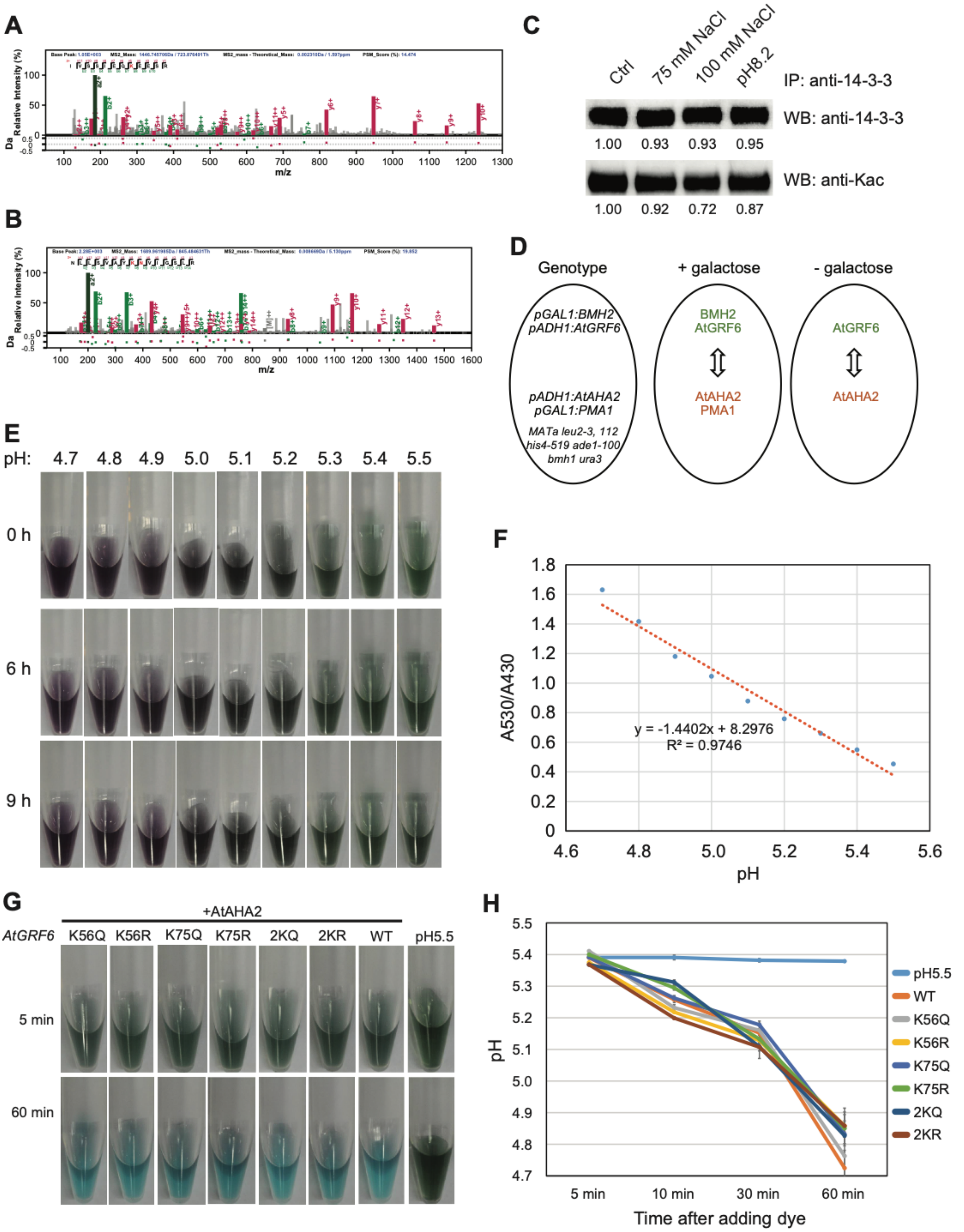

Supplemental Figure 6 Acetylation of Lys56 in GRF6 negatively regulates alkaline tolerance response. (**A**) and (**B**) MS spectra showing the peptide NLLSVAYKNVIGSLR (A) and IVSSIEQKEESR (B) containing acetylated lysine at Lys56 and Lys75 of GRF6, respectively. (**C**) Western blot showing the protein and acetylation level of immunoprecipitated 14-3-3 proteins. (**D**) Diagrams of the genotype and the 14-3-3 proteins and proton ATPases expressed in the modified RS72v strain. (**E**) Colors of the methyl purple solutions under different pH. The colors are stable for at least 9 hours after the dye is added. (**F**) Standard curve for pH

measurement. The green color of methyl purple has spectral absorption at 530 nm, and the purple color has spectral absorption at 430 nm. The A530/A430 ratio linearly correlates with the pH from 4.7 to 5.5. (**G**) Methyl purple staining showing the pH changes of yeast cells harboring AtAHA2 and different version of AtGRF6 growing in galactose-containing liquid medium. (**H**) The pH changes of the yeast cells in (G).
